# Establishment and Characterization of a Nude Mouse Model of Subcutaneously Implanted Tumors and Abdominal Metastasis in Gastric Cancer

**DOI:** 10.1155/2017/6856107

**Published:** 2017-04-11

**Authors:** Yin Zhu, Yi Hu, Ming Cheng, Chun-Yan Zeng, Zhen Yang, Xiao-Dong Zhou, Jiang Chen, Nong-Hua Lu

**Affiliations:** ^1^Department of Gastroenterology, The First Affiliated Hospital of Nanchang University, Nanchang, Jiangxi, China; ^2^Department of Orthopedics, The First Affiliated Hospital of Nanchang University, Nanchang, Jiangxi, China; ^3^Department of Oncology, The First Affiliated Hospital of Nanchang University, Nanchang, Jiangxi, China

## Abstract

A mouse gastric cancer model is an important tool for studying the mechanisms of gastric cancer. To establish subcutaneously implanted tumors, MKN-45 cell suspensions and tumor tissues were implanted into the middle of the right armpit of nude mice. To generate an abdominal metastasis model, MKN-45 cell suspensions and tumor tissue homogenates were implanted into the middle of the lower abdomen. We measured the weights of the nude mice and the longest dimension, shortest dimension, thickness, and volume of the tumor. We also analyzed the rate of tumor formation, the time required for tumor formation, and the number and size of abdominal tumors in the mice. The rates of formation of the subcutaneously implanted tumors were 100%, 0%, and 100% in the nude mice inoculated with 2 × 10^7^ cells/mL or 1 × 10^7^ cells/mL of the MKN-45 cell suspension or the tumor tissue homogenate (2 × 10^7^ cells/mL), respectively. The rates of metastatic abdominal tumor formation were 100%, 50%, and 75% in mice inoculated with 5 × 10^7^ cells/mL or 1 × 10^7^ cells/mL of the tumor tissue homogenate or the MKN-45 cell suspension (5 × 10^7^ cells/mL), respectively. We derived tumor tissues and tumor tissue homogenates from nude mice prior to establishing the subcutaneous model of implanted tumors and the abdominal metastasis model of gastric cancer, respectively.

## 1. Introduction

Gastric cancer (GC) is the fourth most common cancer and the third most common cancer-related cause of death worldwide [1]. In China, a country whose population accounts for one-fifth of the world population, GC is the second most commonly diagnosed cancer and the second leading cause of cancer-related death [2]. Multiple factors are involved in gastric carcinogenesis, including *Helicobacter pylori* infection, environmental factors, diet, smoking, and Epstein-Barr virus infection [3]. However, many factors remain unknown or unclear despite the considerable evolution of our knowledge of GC pathogenesis over the years. Surgery is the primary and curative GC treatment method [4]. However, the majority of patients who are diagnosed with GC exhibit stage IV disease at the time of diagnosis. GC patients have low rates of radical resection surgery and short overall survival time (the median overall survival duration is less than 1 year) [5]. Molecular therapies that target a variety of mechanisms may be developed into efficient methods to treat GC. These targeted mechanisms include the regulation of epidermal growth factor, angiogenesis, immune checkpoint blockades, the cell cycle, cell apoptosis, key enzyme levels and c-Met, mTOR, and insulin-like growth factor receptor signaling [6–8].

The establishment of a gastric cancer model in vivo or vitro is an important tool for in-depth studies of the mechanisms of GC and the determination of the efficacy of antibodies that may be useful for GC treatment. Numerous studies of GC mechanisms have been performed using cell lines (e.g., SGC-7901, AGS, and MKN-45 cells) and primary cell cultures. Although cell lines possess some advantages in that they are simple, provide good controls, and have short life cycles, studying cells in vitro is different than studying cancer cells in vivo [9]. To date, a considerable number of researchers have successfully established patient-derived models of gastric carcinoma metastasis in mice [10–12]. However, the tumor formation rates are relatively low in these models (less than 30%), and the use of patient-derived gastric carcinomas introduces heterogeneity and randomness to these studies. In this study, we successfully established nude mouse subcutaneously implanted tumor and abdominal metastasis models for GC. Moreover, we compared the tumor formation rates, growth rates, and the morphologies and histopathologies of GC tumors created using two different inoculation methods (e.g., cell suspensions and tumor tissues).

## 2. Materials and Methods

### 2.1. Cell Line

The human gastric carcinoma cell line MKN-45 was kindly provided by Dr. Daiming Fan of Xi-Jing Hospital of Digestive Diseases, Xi'an, Shanxi, China. The MKN-45 cells were cultured in Dulbecco's Modified Eagle's Medium (DMEM) supplemented with 10% fetal bovine serum (FBS), 100 U of penicillin, and 100 *μ*g/mL of streptomycin (Gibco of Thermo Fisher Scientific Inc., Waltham, MA, USA) at 37°C in an atmosphere containing 5% CO_2_.

### 2.2. Nude Mice

Six- to eight-week-old specific pathogen-free female BALB/c nude mice were provided by a national rodent seed center (China Laboratory Animal Center of the Pharmaceutical and Biological Products Institute) and maintained in an isolated clean room held at a regulated temperature (25 ± 2°C) and humidity (approximately 40–50%). The mice were housed under a 12 h/12 h light/dark cycle and fed ad libitum with rodent diet and water. All protocols were approved and performed according to the guidelines of the Ethics Committee of The First Affiliated Hospital of Nanchang University.

### 2.3. Establishment of a Subcutaneously Implanted Tumor Model

The nude mice were divided into three groups (4 nude mice per group). The nude mice were intraperitoneally injected with an anesthetic (a mixture of xylidinothiazoline, ethylenediaminetetraacetic acid, dihydroetorphine hydrochloride, and haloperidol, 0.5 mg/kg).

1. Inoculation method for the MKN-45 cell suspensions. The cells were collected in the exponential phase and digested into single-cell suspensions. Then, the concentration of these single-MKN-45 cell suspensions was adjusted to 1 × 10^7^ cells/mL or 2 × 10^7^ cells/mL. Anesthetized nude mice were disinfected with 75% alcohol and then inoculated with 200 *μ*L of one of the two cell suspensions in the middle of the right armpit. The mice were disinfected again and placed in laminar air flow rack while their physical signs were monitored.
2. Inoculation method for tumor tissues. The primary implanted tumors in mice inoculated with MKN-45 GC cells were successfully collected once the diameter of the primary metastasis had grown to 1 cubic centimeter and implanted into Allogen under sterile conditions. To collect the tumors, the nude mice were sacrificed using cervical dislocation. The region over the tumor was disinfected, and the subcutaneously implanted tumors were removed using eye scissors and ophthalmic forceps. The tumor tissues (cut to 2 × 2 × 2 mm) were implanted into the middle of the right armpit of new nude mice using ophthalmic forceps. The nude mice were placed in a laminar air flow rack, and their physical signs were observed.

### 2.4. Establishment of an Abdominal Metastasis Model

The nude mice were divided into three groups (4 nude mice per group).

1. Abdominal inoculation method for cell suspensions. The cells were collected in the exponential phase and digested to form single-cell suspensions. Then, the concentration of a single-MKN-45 cell suspension was adjusted to 5 × 10^7^ cells/mL. Anesthetized nude mice were disinfected with 75% alcohol and then inoculated with 200 *μ*L of the cell suspension in the middle of the lower abdomen. The mice were disinfected again and placed in a laminar air flow rack. Their physical signs were monitored.
2. Inoculation method for tumor tissue homogenates. When the diameters of the primary implanted tumors had grown to 1 cubic centimeter, the nude mice were sacrificed using cervical dislocation. The mice were disinfected, and the subcutaneously implanted primary tumors were dissected using eye scissors and ophthalmic forceps. The cells were digested into single-cell suspensions (1 × 10^7^ cells/mL or 5 × 10^7^ cells/mL). Anesthetized nude mice were disinfected using 75% alcohol and inoculated with 200 *μ*L of a cell suspension (1 × 10^7^ cells/mL or 5 × 10^7^ cells/mL) in the middle of the lower abdomen. Then, the mice were disinfected again and placed in a laminar air flow rack, and their physical signs were monitored.

### 2.5. Index

1. We measured the weights of the nude mice and the longest axis (*a*), shortest axis (*b*), and thickness (*c*) of the tumor tissues. The tumor volume (*V*) was evaluated using the formula *V* = *πabc*/6 [13]. Growth curves were constructed using the average tumor volumes.
2. The rate of tumor formation, the progression time to tumor formation, and the numbers and sizes of the abdominal tumor tissues were measured.
3. A pathological examination of the implanted and metastatic tumor model mice was conducted using hematoxylin and eosin (H&E) staining.

### 2.6. Data Analysis

Continuous variables are presented as the means and standard deviations. Categorical variables are presented as numbers and percentages and have been analyzed using Fisher's exact test. A *p* value less than 0.05 was considered significant. All data were analyzed using the SPSS, version 19.0 software for Windows (SPSS, Chicago, IL, USA).

## 3. Results

### 3.1. The Formation of Subcutaneously Implanted Tumors

Tumors were observed in nude mice (4/4, 100%) subcutaneously inoculated with the more concentrated MKN-45 cell suspension (200 *μ*L, 2 × 10^7^ cells/mL). The tumor formation rate was also 100% (4/4) in the nude mice inoculated with the tumor tissue homogenate. No subcutaneous swelling or tumors were observed in the nude mice inoculated with the less concentrated MKN-45 cell suspension (200 *μ*L, 1 × 10^7^ cells/mL).

### 3.2. The Growth of Subcutaneously Implanted Tumors

**Figure 1:**
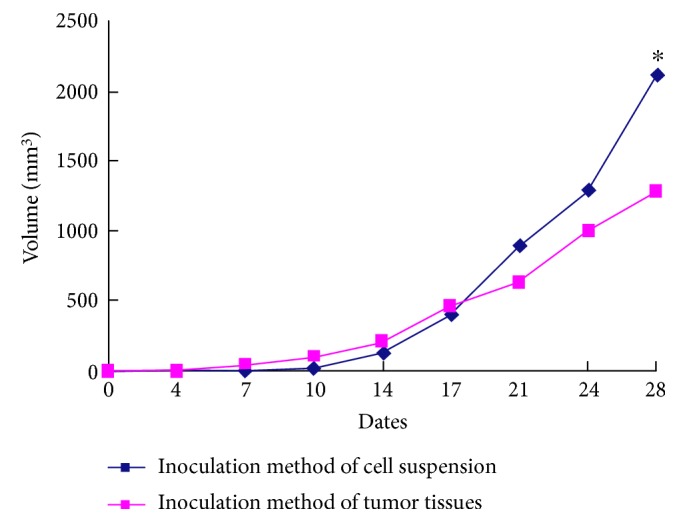
The growth curves of tumors grown using two methods. ^∗^*p* < 0.05 versus the group inoculated with tumor tissues.

The mean time required for the tumor diameters to grow to 0.5 cm was shorter in the group of mice inoculated with the tumor tissue (7 ± 0.5 days) than that in the group inoculated with the cell suspension (10 ± 1.5 days). In the group inoculated with the cell suspension, the subcutaneously implanted tumors grew slowly for two weeks after implantation; then, the volumes of the tumors significantly increased after two weeks. The mean tumor volume in these mice was 2107.5 ± 897.5 mm^3^ after 28 days. The homogeneity of these tumors was poor, with each tumor exhibiting significant differences from the other tumors. In the group inoculated with tumor tissues, the tumors formed during the early stage and then grew at a relatively constant rate to achieve a mean volume of 1272.5 ± 312.5 mm^3^ after 28 days (Figure 1). These tumors showed substantial homogeneity. Taken together, these results indicate that the inoculation method for tumor tissues should be optimized prior to establishing a subcutaneously implanted tumor model in nude mice.

### 3.3. The Tumor Morphology in Each Group

**Figure 2:**
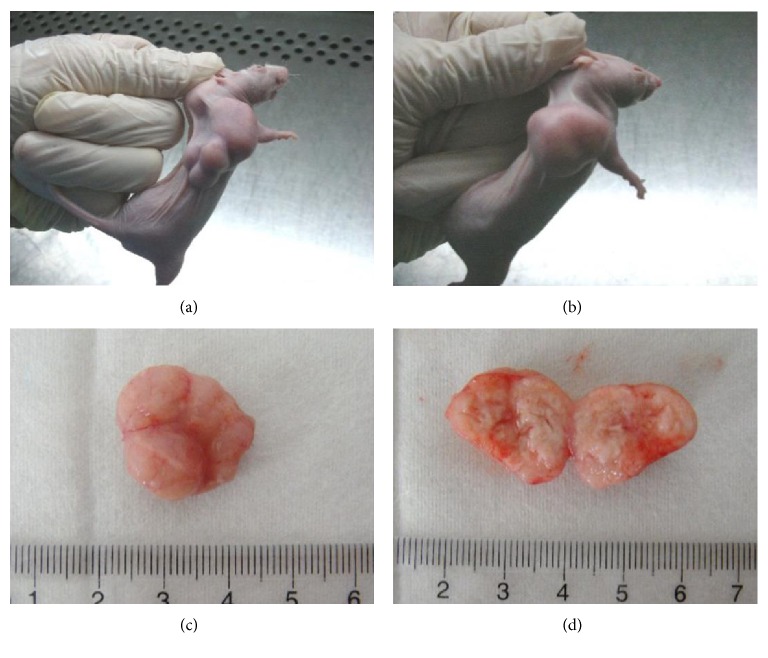
A subcutaneously implanted tumor model of GC and the morphology of tumors from subcutaneously implanted cells and tissues. (a) Cell suspension inoculation method, (b) tumor tissue inoculation method, and (c and d) gross morphologies of the tumors in the groups inoculated with the tumor tissue method.

In the group inoculated with the cell suspension, the tumor morphologies were irregular, and multiple tumor nodules were observed. In the group inoculated using tumor tissues, the tumors were singletons, regular, and oval-shaped. In both groups, the tumors were fleshy pink, exhibited clear boundaries, and formed multiple microvascular structures on the tumor surfaces. The tumors were fish-shaped and firm. Necrosis was observed in the center of the tumors (Figure 2).

### 3.4. Histopathology of the Tumors in Each Model

**Figure 3:**
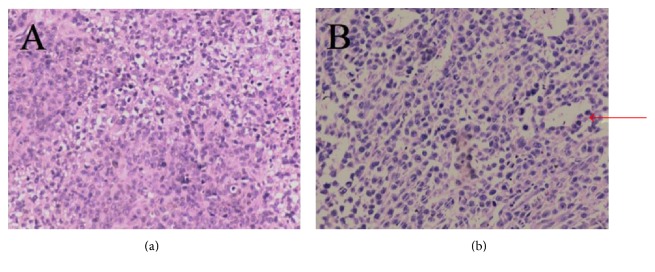
H&E staining of subcutaneously implanted tumor tissues in nude mice. (a) Cell suspension inoculation method and (b) tumor tissue inoculation method. Arrow: adenoid structure.

The histopathology of the subcutaneously implanted tumors was similar between the two groups (e.g., inoculation using a cell suspension or tumor tissue). The tumor capsule was complete, multiple irregular cells were present, the cells exhibited hyperchromatic nuclei and obvious heteromorphism, less intercellular connective tissue was present, and multiple necrotic cells were observed. The arrangement of the tumor cells was chaotic in the group inoculated with the cell suspension, and no obvious adenoid structures were observed in this group. However, a portion of the cancer cells were arranged in adenoid formation in the group inoculated with tumor tissue (Figure 3).

### 3.5. A Tumor Abdominal Metastasis Model

**Figure 4:**
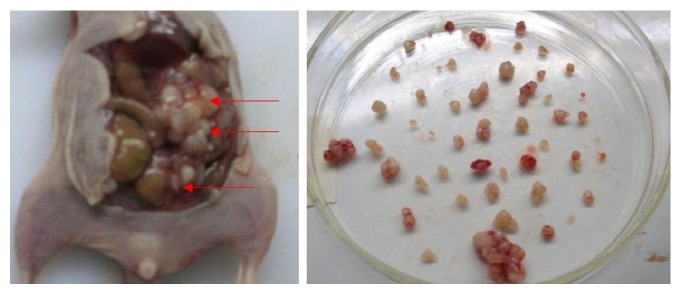
Disseminated abdominal lesions were induced in mice inoculated with cell suspensions. Arrow: disseminated lesions.

Disseminated abdominal lesions were observed in 3 out of 4 nude mice (75%) inoculated with the MKN-45 cell suspension (200 *μ*L, 5 × 10^7^ cells/mL). The tumor nodules differed from one another. Although the diameters of most of the nodules ranged from 1–5 mm, some of the tumor nodules were up to 15 mm in size. Disseminated abdominal lesions were observed in 2 of the 4 nude mice (50%) inoculated with the tumor tissue homogenate with the lower cell concentration (200 *μ*L, 1 × 10^7^ cells/mL). In these mice, the tumor nodules were nonhomogeneous, and the diameters of most nodules ranged from 1–10 mm (Figure 4). Disseminated abdominal lesions were observed in 4 out of 4 (100%) nude mice inoculated with the tumor tissue homogenate with the higher cell concentration (200 *μ*L, 5 × 10^7^ cells/mL); the tumor nodules in these mice were homogeneous, with diameters ranging from 1–5 mm.

## 4. Discussion

Several factors and processes are involved in gastric carcinogenesis. Although the early rate of diagnosis and comprehensive therapies for GC have improved in recent years, the mortality rate of GC remains high, and the five-year survival rate remains low. Establishing a subcutaneously implanted tumor model is an important tool for studies of the mechanisms involved in GC. The dissemination of primary and implanted tumors in the abdomen after surgery accounts for GC recurrence after surgery and is an important factor that influences the five-year survival rate. The establishment of an abdominal metastasis model is an essential tool that can be used to illustrate the metastatic mechanisms underlying tumor formation and dissemination in the abdomen and to contribute to the identification of new therapeutic regimens to treat GC.

MKN-45 cells are low-differentiated glandular cancer cells that exhibit high malignancy. In our study, we successfully established a subcutaneously implanted GC tumor model in nude mice by applying two inoculation methods (cell suspensions and tumor tissues). Moreover, we compared the advantages and disadvantages of each method. The results showed that the tumor formation rate was 100% (4/4) both in nude mice inoculated with the MKN-45 cell suspension (200 *μ*L, 2 × 10^7^ cells/mL) and in those inoculated with tumor tissue. Compared to the group inoculated using cell suspension, the growth of the tumors was uniform, the tumor shapes were regular, and the tumor sizes were consistent in the group inoculated using tumor tissue, which was prior to experimental study. This method also reduced the process required to culture GC cells, reduced experimental costs, and simplified the experimental procedures, all of which would support the larger-scale establishment of a similar subcutaneously implanted tumor model. The first step in our method used to inoculate mice with tumor tissues was to subcutaneously inject the MKN-45 cell suspension, which led to the formation of tumors. In practice, this process was used to screen the injected tumor cells because we observed heterogeneity in the tumor cells. The screened tumor cells were active and formed subcutaneous tumors. Therefore, the time required to form tumors was shortened in the group inoculated with tumor tissues when we used the active screened tumor cells. The method inoculated with the MKN-45 cell suspension was simple and led to a high rate of tumor formation. However, the tumors in these mice grew more slowly in the early stage, took longer to form tumors, and formed tumors with irregular shapes and inconsistent sizes. These characteristics indicate that a prolonged experimental period is required, which was not prior to experimental study. Moreover, no subcutaneous swelling and tumors were observed in the nude mice subcutaneously inoculated with the less concentrated MKN-45 cell suspension (200 *μ*L, 1 × 10^7^ cells/mL).

A previous study [14] indicated that tumors formed in the abdomen when mice were inoculated with 1 × 10^6^ cells/mL. In that study, the authors observed heterogeneity in the tumor cells and found that transforming growth factor-*β*, integrin, and other adhesion molecules played important roles in the processes involved in the formation of nodules transferred to the peritoneum [15, 16]. In our study, we subcutaneously inoculated nude mice in the abdomen with tumor tissue homogenates (1 × 10^7^ cells/mL or 5 × 10^7^ cells/mL) or a cell suspension (5 × 10^7^ cells/mL) (200 *μ*L each). The results showed that the rate at which tumors formed was 50% and that inconsistent nodules were observed in the group inoculated with the low cell concentration tumor tissue homogenate (1 × 10^7^ cells/mL). The rate at which tumors formed in the group inoculated with the cell suspension (5 × 10^7^ cells/mL) was 75%, and inconsistent nodules were also observed in this group. The rate at which tumors formed in the group inoculated with the high cell concentration tissue homogenate (5 × 10^7^ cells/mL) was 100%, and consistent nodules were observed in this group. Therefore, the establishment of a subcutaneously implanted tumor model using tumor tissue homogenates as the inoculation method (5 × 10^7^ cells/mL) is appropriate.

## 5. Conclusions

We successfully established a GC model of subcutaneously implanted tumors and an abdominal metastasis model in nude mice. We evaluated the method to inoculate the tumor tissues prior to establishing the subcutaneously implanted tumor model in addition to evaluating the method for the inoculation of tumor tissue homogenates to optimize this abdominal metastasis model. Thus, the establishment of this model can be used to study the mechanisms underlying GC and to screen and identify therapeutic drugs to treat GC.
